# Nanoparticle Delivery of Fidgetin siRNA as a Microtubule-based Therapy to Augment Nerve Regeneration

**DOI:** 10.1038/s41598-017-10250-z

**Published:** 2017-08-29

**Authors:** Timothy O. Austin, Andrew J. Matamoros, Joel M. Friedman, Adam J. Friedman, Parimala Nacharaju, Wenqian Yu, David J. Sharp, Peter W. Baas

**Affiliations:** 1 Department of Neurobiology and Anatomy, Drexel University College of Medicine, Philadelphia, PA 19129 USA; 2 Department of Physiology and Biophysics, Albert Einstein College of Medicine, Bronx, NY 10461 USA; 3 Department of Dermatology, George Washington School of Medicine and Health Sciences, Washington, DC 20037 USA

## Abstract

Microtubule-stabilizing drugs have gained popularity for treating injured adult axons, the rationale being that increased stabilization of microtubules will prevent the axon from retracting and fortify it to grow through inhibitory molecules associated with nerve injury. We have posited that a better approach would be not to stabilize the microtubules, but to increase labile microtubule mass to levels more conducive to axonal growth. Recent work on fetal neurons suggests this can be accomplished using RNA interference to reduce the levels of fidgetin, a microtubule-severing protein. Methods to introduce RNA interference into adult neurons, *in vitro* or *in vivo*, have been problematic and not translatable to human patients. Here we show that a novel nanoparticle approach, previously shown to deliver siRNA into tissues and organs, enables siRNA to gain entry into adult rat dorsal root ganglion neurons in culture. Knockdown of fidgetin is partial with this approach, but sufficient to increase the labile microtubule mass of the axon, thereby increasing axonal growth. The increase in axonal growth occurs on both a favorable substrate and a growth-inhibitory molecule associated with scar formation in injured spinal cord. The nanoparticles are readily translatable to *in vivo* studies on animals and ultimately to clinical applications.

## Introduction

The regenerative capacity of injured adult axons is limited, particularly in the central nervous system. Injured axons tend to degenerate. If they do regrow, they encounter obstacles such as scar tissue and inhibitory molecules, and their growth rates simply do not match that of a juvenile axon^1, 2^. In recent years, the regeneration community has been intrigued by the idea of microtubule-stabilizing drugs as a therapy to augment nerve regeneration. Although microtubules in adult axons are already more stable than in juvenile axons, the premise is that perhaps the injured adult axon would retract less, grow better, and power its way through inhibitory environments if its microtubules were even more stable. Encouraging results with microtubule-stabilizing drugs have been obtained with preclinical rodent models for spinal cord injury^3, 4^. However, some of the results have been difficult to reproduce, with limited benefits more attributable to drug effects on scar tissue-forming cells rather than on neurons^5^. In addition, the logic of microtubule stabilization as a therapy has been questioned for various reasons, including negative effects that could outweigh positive benefits^6, 7^. For example, an individual microtubule in the axon consists of a stable domain and a labile domain, with each domain having important work to do^8^. Hence, stabilizing the labile domain could incapacitate a portion of that work.

Rapidly growing axons tend to have a higher proportion of labile microtubule mass^7^. For this reason, our premise is that we can augment nerve regeneration by increasing labile microtubule mass in the axon. We posit that we can do so by reducing the levels of fidgetin, a microtubule-severing protein that normally exists to pare back the labile microtubule mass of the axon^9^. To apply this strategy to adult neurons in animals and human patients, we need a method of delivery of siRNA that is effective, safe, and minimally invasive. Here we propose to use a relatively new nanoparticle delivery system, which we have tested on cultures of adult rat dorsal root ganglion (DRG) neurons, a well-accepted *in vitro* model for spinal cord injury. Based on a hydrogel/sugar glass composite, our siRNA delivery platform is a hybrid nanoparticle capable of encapsulating and controllably releasing a broad range of therapeutically relevant materials ranging from gaseous nitric oxide to larger macromolecules such as chemotherapeutic agents and phosphodiesterase inhibitors^10^. The nanoparticles have been shown to be capable of delivering siRNA to tissues and organs^10^.

## Results

**Figure 1:**
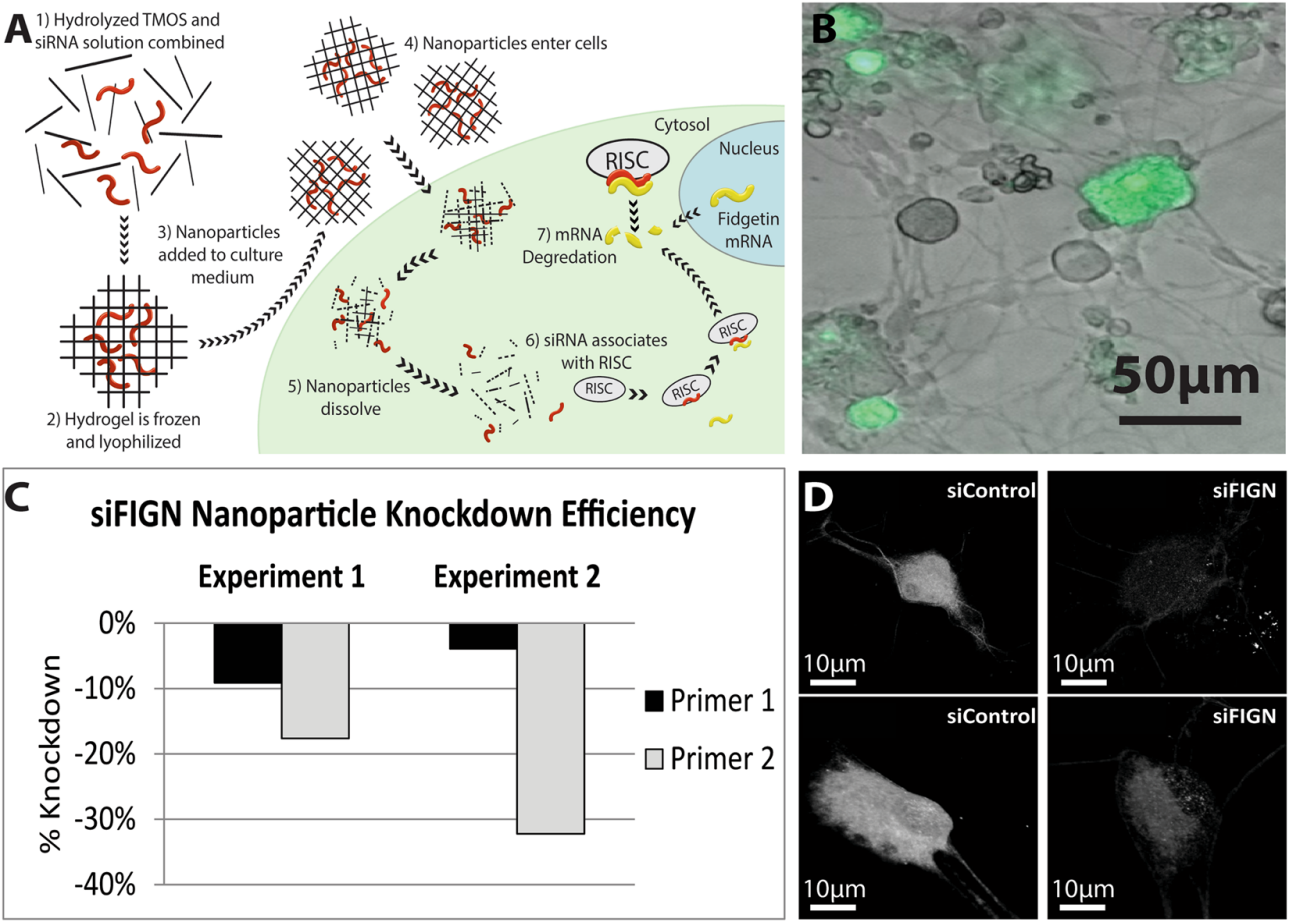
Validation of siRNA nanoparticles in adult DRG primary cultures. (**A**) Schematic detailing the production of siRNA hydrogel nanoparticles, nanoparticle entry and release into the cell, and siRNA targeting fidgetin mRNA. (**B**) Nanoparticles containing alexa488 were added to culture medium and a representative merged image of bright-field and green-channel fluorescence is shown; large diameter neurons displayed more uptake of nanoparticles compared to other cells. Uptake is more visible in clusters of neuronal cell bodies. (**C**) qRT-PCR was performed on samples of RNA from siFidgetin-nanoparticle treated DRG primary cultures; data from two separate primers are shown in the bar graph. (**D**) Representative fidgetin IF images (confocal z-stack images) of DRG neuronal cell bodies indicating knockdown of fidgetin from neurons treated with siRNA nanoparticles.

When added to culture medium, the siRNA-encapsulated nanoparticles readily cross the cell membrane and dissolve, releasing the siRNA into the cytoplasm to interact with the RNA-induced silencing complex (Fig. 1A-schematic). Preliminary studies with nanoparticles conjugated to a fluorescent dye demonstrated that the nanoparticles enter neurons in adult DRG cultures (Fig. 1B), which is not surprising given that they have previously been shown to effectively enter intact tissues and organs^10^. It was our impression that neurons, especially ones with larger cell bodies, took up the nanoparticles better than the smaller and flatter cells in the culture. qPCR revealed that some batches of nanoparticles failed to knock down fidgetin (relative to control non-specific siRNA nanoparticles), while other batches knocked down mRNA by 25–50% (Fig. 1C). The former batches, which presumably were defective at some point in the preparative procedure, were discarded. Knockdown of fidgetin protein (relative to control siRNA nanoparticles) with the qPCR-effective batches specifically in the neurons of the culture was confirmed by immunofluorescence (IF) for fidgetin (Fig. 1D). A reduction of IF signal intensity was particularly notable in neurons, perhaps due to their more efficient uptake of the nanoparticles.

**Figure 2:**
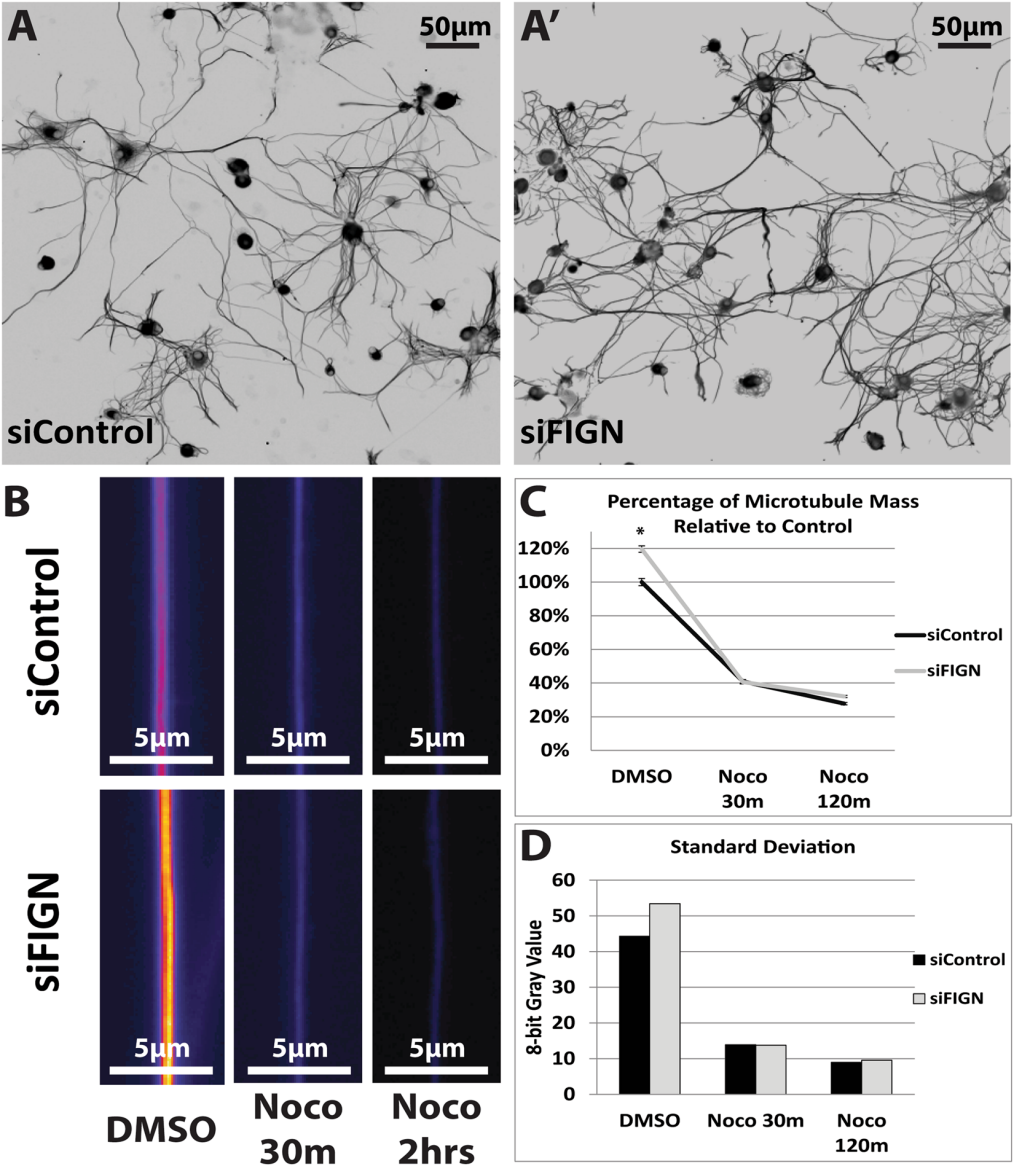
Microtubule mass is increased in the axons of adult DRG neurons as a result of fidgetin knockdown. **(A**) Representative images of neuronal β-III-tubulin IF-staining show more extensive axonal outgrowth after fidgetin knockdown compared to control, and also show denser microtubule mass within axons, as indicated by more intense IF-staining. Shown are inverted images with black and white reversed for enhanced clarity. (**B**) Representative IF displayed with quantitative pseudo color (standard fire-scale where purple is the least intense, white is the most intense, with shades of orange and red between them) to further accentuate the increased microtubule mass in axons of siFidgetin nanoparticle-treated neurons. Additionally, (**B**) shows that after 30 minutes or 2 hours in nocodazole, there is no discernable difference in microtubule levels between fidgetin and control siRNA. (**C**) Quantification of axonal tubulin IF shows a 19% increase in siFidgetin nanoparticle treated axons compared to controls, Mann-Whitney U test, *p < 0,005. (**D**) Standard deviation of tubulin IF 8-bit grayscale values, higher when the labile fraction is intact and lower when it has been depolymerized, is consistent with the labile domains of the microtubules being more dynamic than the stable domains.

Cultures exposed to fidgetin siRNA nanoparticles were markedly different in morphology, as assessed by phase-contrast microscopy, from cultures exposed identically to control siRNA nanoparticles, the latter of which were indistinguishable in appearance from cultures without siRNA treatment (not shown). Qualitatively, the cultures treated with fidgetin siRNA nanoparticles displayed a greater amount of axonal mass than controls, which was also apparent in cultures IF-stained for neuron-specific β-III-tubulin to reveal microtubules (Fig. 2A and A’).

### Nanoparticle delivery of fidgetin siRNA increases labile microtubule mass in the axons of cultured rat adult DRG neurons

Before using sparser cultures to quantify the morphological differences, we used denser cultures to ascertain microtubule levels and stability, as per our earlier work on fetal cortical neurons. Adult DRG cultures are notoriously heterogeneous, consisting of three classes of neurons as well as non-neuronal cells such as satellite cells and fibroblasts. Neurons are not as numerous compared to the other cell types, making Western blotting problematic as a means to assess the status of the microtubules specifically in the neurons. We suspected that the more modest level of fidgetin knockdown achieved with the nanoparticle approach would result in a more modest elevation in microtubule mass than in our earlier study on fetal neurons, in which we were able to achieve near complete fidgetin knockdown by introducing the siRNA by nucleofection^9^. For these reasons, we endeavored an IF approach that would acquire data from virtually every neuron in the culture, with sample numbers of roughly 600 for each experimental condition. We reasoned that a more modest effect would likely require a higher sample number to achieve statistical significance, especially in light of the greater heterogeneity of DRG neurons compared to cortical neurons.

Three separate dissections were performed to obtain primary cultures that were then cultured for three days with either control siRNA or fidgetin siRNA nanoparticles. Experiments were conducted in duplicate. Axonal microtubule fluorescence values were not normally distributed (Shapiro-Wilk test, p < 0.005 per group) for control siRNA treated neurons (n = 1,726) with a skewness of 2.415 (standard error = 0.059) and kurtosis of 7.706 (standard error = 0.118), or for fidgetin siRNA treated neurons (n = 1,888) with a skewness of 1.949 (standard error = 0.056) and kurtosis of 4.409 (standard error = 0.113). The data are positively skewed for both groups and therefore a non-parametric comparison of medians was performed. A Mann-Whitney U test was conducted to measure differences in microtubule fluorescence between control siRNA and fidgetin siRNA treated neurons. Distributions of microtubule fluorescence values for control siRNA and fidgetin siRNA were similar upon visual inspection, permitting a comparison of medians. Median fluorescence value was statistically significantly higher in fidgetin siRNA treated neurons (43.03) than in control siRNA treated neurons (36.91), U = 1,850,657.5, z = 1,850,657.5, p < 0.005.

As with the previous studies on fetal cortical neurons, we included a 30-minute treatment with nocodazole as well as a 2-hour treatment, in order to assess whether any increase in microtubule mass that we might document is primarily of the labile or stable microtubule fraction (Fig. 2B). In the adult DRG neurons treated with fidgetin siRNA (56.28 ± 42.50), there was a 19% increase in mean microtubule mass relative to control siRNA (47.53 ± 36.56) per unit area of axon (Fig. 2C), which is about a third as much of an increase as we previously reported of the fetal cortical neurons with more complete fidgetin knockdown. After 30 minutes of nocodazole treatment, the microtubule levels in cultures treated with fidgetin siRNA were indistinguishable from those in cultures treated with control siRNA, which was also the case in our earlier study on fetal cortical neurons; therefore, no statistical testing was conducted. These results indicate that the microtubule mass added to the axon as a result of fidgetin depletion is predominantly or entirely labile, because very little of the stable microtubule fraction would be diminished after 30 minutes of drug treatment. Interestingly, the standard deviation for the total microtubule mass was notably higher than for that remaining after 30 minutes of drug treatment (Fig. 2D), which is consistent with the labile component of the microtubule mass being highly dynamic and the stable component being much less dynamic.

**Figure 3:**
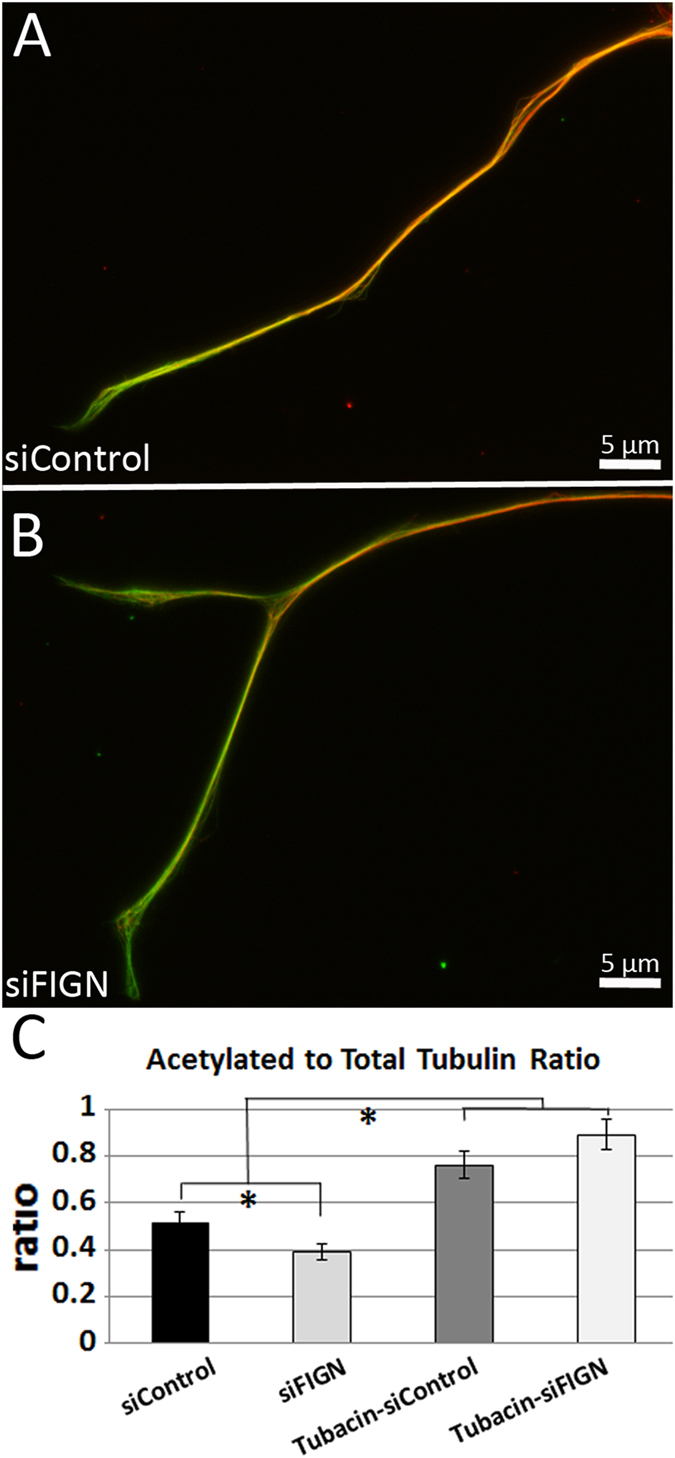
Fidgetin knockdown decreases the ratio of acetylated to total tubulin in the axons of DRG neurons. (**A**,**B**) IF-staining for β-III-tubulin (green) and acetylated tubulin (red), shown as overlays of the two colors. Ratio of acetylated tubulin to β-III-tubulin (total tubulin) was significantly decreased as a result of fidgetin knockdown (**A**,**B**,**C**). The acetylated/total tubulin ratio of both treatments increased after tubacin treatment, with the control and fidgetin siRNA becoming statistically indistinguishable.

Finally, the ratio of acetylated to total tubulin was measured in control siRNA and fidgetin siRNA treated neurons, with and without tubacin, a histone deacetylase inhibitor that increases microtubule acetylation. Without tubacin, the ratio of acetylated to total tubulin relative to control siRNA (Fig. 3A) was lower in axons of fidgetin siRNA treated neurons (Fig. 3B). In the presence of tubacin, the ratio was heightened for both. Data (shown in Fig. 3C) are mean ± standard error. There were 28 control siRNA and 29 fidgetin siRNA axons measured. There were no outliers and the data were normally distributed per group, as assessed by histogram analysis and Shapiro-Wilk test (p > 0.05 per group). Levene’s Test for Equality of Variance confirmed that variance was equal (p = 0.257). There was a statistically significant difference in mean acetylation ratios between control and fidgetin siRNA treatment groups, t(55) = 2.389, p = 0.020. Mean acetylation ratio for control siRNA treated axons (0.52 ± 0.23) was significantly higher than mean fidgetin siRNA treated axons (0.39 ± 0.18). Our results are consistent with fidgetin knockdown resulting in the addition of labile microtubule mass, because the ratio of acetylated to total tubulin is expected to be lower upon the addition of labile microtubules^9^.

### Fidgetin knockdown results in increased axonal outgrowth in a manner dependent upon unacetylated tubulin

In order to quantify the morphological effects of the fidgetin knockdown, we grew a set of cultures at a 4-fold sparser density. For these experiments, we also treated some of the cultures with tubacin. In our previous studies on fetal cortical neurons, tubacin prevented fidgetin knockdown from increasing axonal length, which is consistent with fidgetin targeting labile domains of microtubules via a preference for unacetylated tubulin^9^. We set out to determine if the same effect is observed in primary DRG cultures supplemented with fidgetin siRNA nanoparticles, as opposed to nucleofection of siRNA.

**Figure 4:**
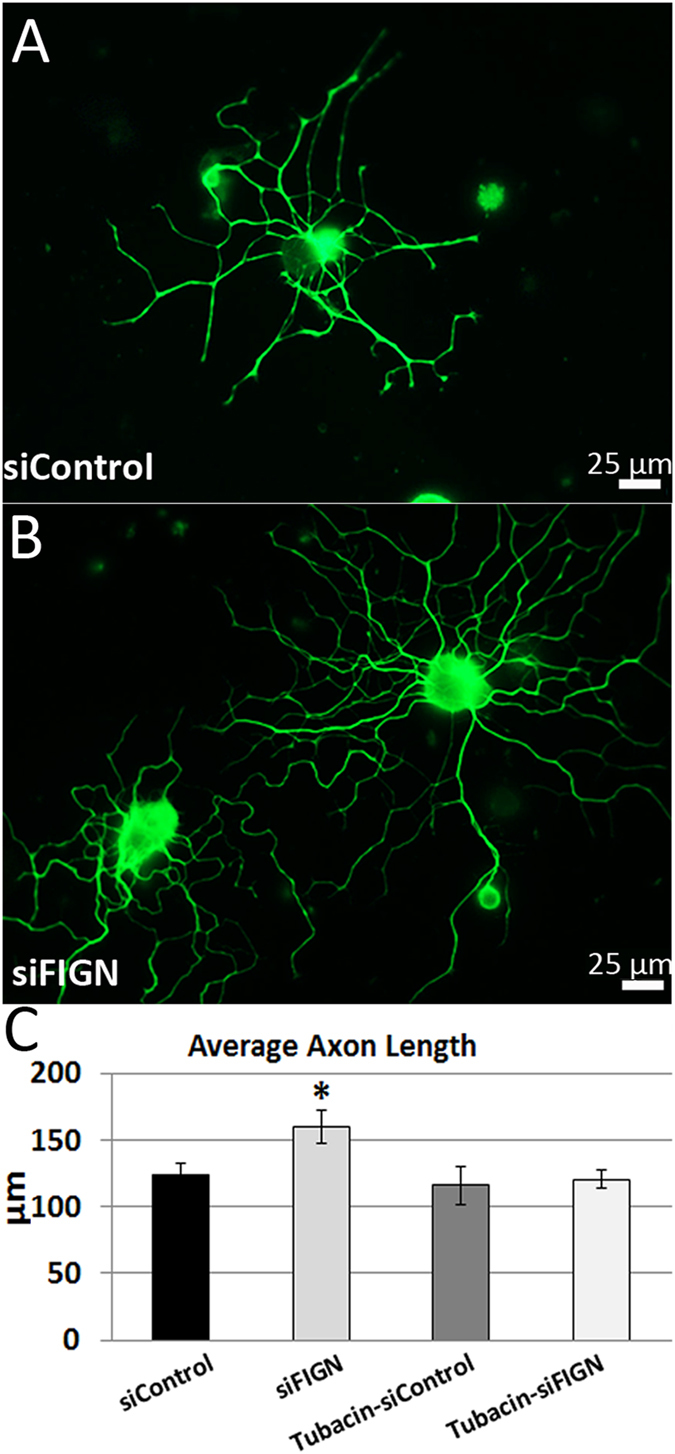
Fidgetin knockdown positively affects axonal outgrowth in a manner dependent upon unacetylated tubulin. Cultured adult DRG cells were treated with siControl (**A**) or siFidgetin nanoparticles (**B**) in combination with vehicle (DMSO) or tubacin. Shown in the panels is IF-staining for β-III-tubulin. Fidgetin knockdown results in a significant increase in average axonal growth compared to control, while tubacin-siFidgetin showed no improvement over tubacin alone (**C**).

Data for axonal length were not normally distributed, as assessed by Shapiro-Wilk Test (p < 0.005). Control siRNA treated axonal length (n = 222) had a skewness of 2.283 (standard error = 0.163) and kurtosis of 7.066 (standard error = 0.325). Fidgetin siRNA treated axonal length (n = 221) had a skewness of 3.444 (standard error = 0.164) and kurtosis of 18.238 (standard error = 13.980). Tubacin treated control siRNA axonal length (n = 114) had a skewness of 4.986 (standard error = 0.226) and kurtosis of 34.048 (standard error = 0.449). Tubacin treated fidgetin siRNA axonal length (n = 405) had a skewness of 4.986 (standard error = 0.121) and kurtosis of 38.187 (standard error = 0.242). The data are positively skewed for all groups and therefore a non-parametric comparison of medians was performed. A Mann-Whitney U test was run to determine if there were differences between the control and fidgetin siRNA axon length. Distributions of axonal length for control and siRNA treatment groups were similar based on visual inspection. Median axonal length was statistically significantly higher for fidgetin siRNA treated axons (95.4 µm) than control siRNA treated axons (79.04 µm), U = 21,828.5, z = −2.006, p = 0.045. Fidgetin knockdown relative to control siRNA (Fig. 4A) resulted in increases in average axon length (Fig. 4B). As in our previous study on fetal cortical neurons^9^, none of these parameters were different between control siRNA and fidgetin siRNA in the presence of tubacin. These results (data shown in Fig. 4C) are consistent with the effects of fidgetin knockdown on axonal growth being dependent upon the acetylation status of the microtubules.

All of the results presented thus far are consistent with the siRNA entering neurons in the culture, reducing fidgetin levels and producing the predicted results on microtubules and neuronal morphology (on the basis of our previous studies)^9^. This provides confidence that the nanoparticle approach is an effective means for transfection of siRNA into adult neurons that are difficult to transfect by traditional means (i.e. nucleofection and lipofectamine have poor transfection efficiency for DRG neurons), and that the same principles of fidgetin knockdown previously reported with fetal neurons apply to adult neurons.

### Fidgetin knockdown promotes axonal growth on non-permissive substrate

**Figure 5:**
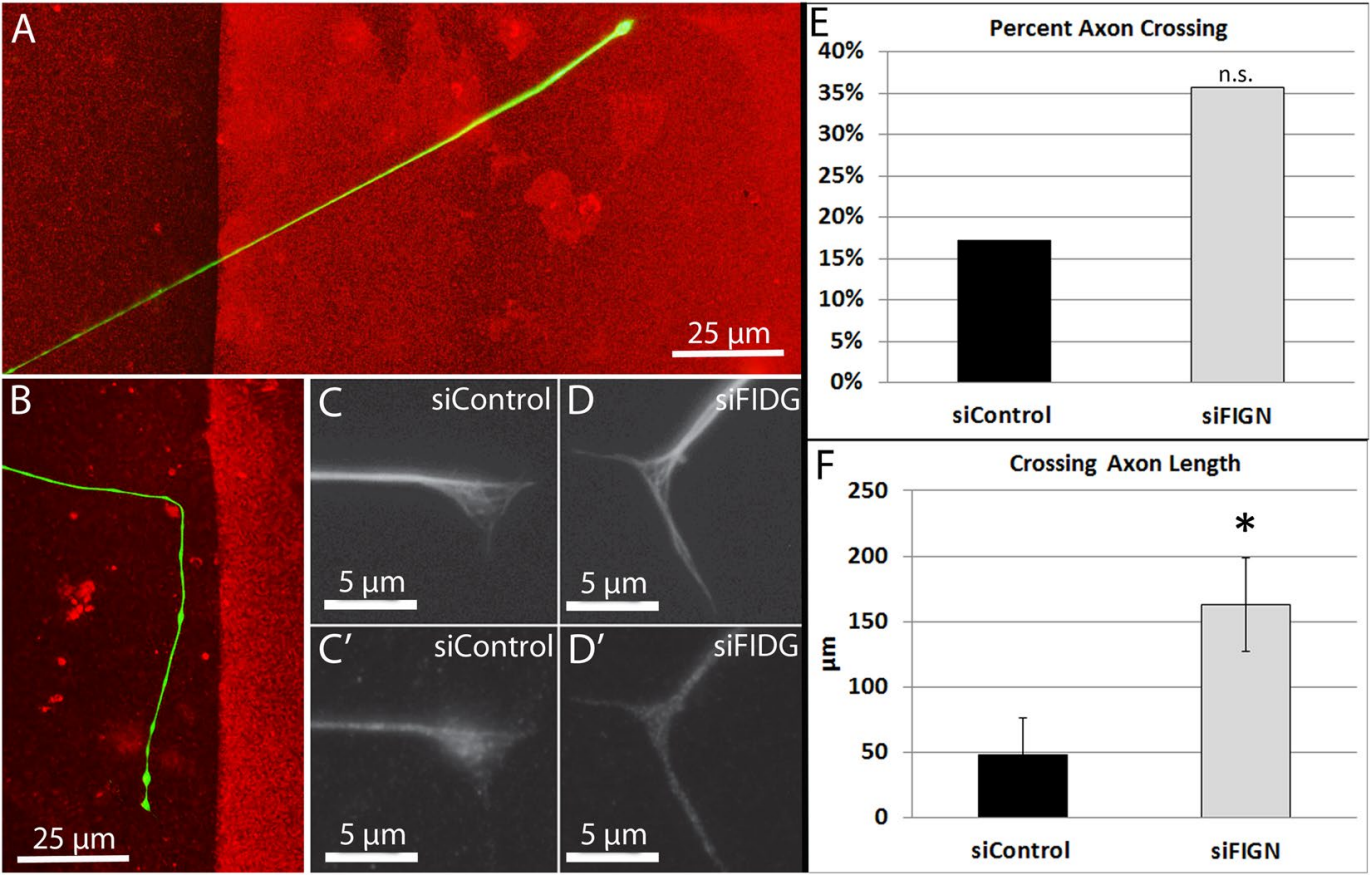
Effects of fidgetin knockdown on axonal growth onto an inhibitory substrate. Cultured adult DRG neurons growing on laminin and treated with either fidgetin or control siRNA nanoparticles were challenged with an aggrecan border. Cultures were IF-stained for β-III-tubulin (green) and aggrecan (red), as shown in panels (**A**,**B**) or IF-stained for β-III-tubulin and fidgetin, as shown in panels (**C**,D’). Axons were scored as a “cross” (**A**) or “no-cross” (**B**). Images were brightened above saturating levels to aid in border identification. (**C**,**D**) Growth cones of β-III IF-staining for siControl- and siFidgetin-treated cultures, respectively. Growth cones with siFidgetin treatment display elongation. (C’,D’) Fidgetin IF-staining for siControl- and siFidgetin-treated growth cones, respectively. (**E**) No significant difference was found in percent axonal crossing between the two groups (Chi-square, p > .05). However, among those axons that did cross, axonal growth was significantly greater (p < .05) in fidgetin siRNA cultures compared to control (**F**).

Axonal regeneration in the adult central nervous system is not only a matter of axons growing faster in permissive environments, but also crossing into growth-inhibitory environments and beyond. The standard cell culture method for testing the capacity of a treatment regime to assist in this regard is to challenge axons to cross from a favorable polylysine-laminin substrate onto a stripe consisting of laminin together with aggrecan, a growth inhibitory protein associated with the glial scar tissue that develops in response to nerve injury. When axons growing on the favorable substrate encounter the aggrecan border, most turn away from their original projection path to avoid crossing onto the aggrecan (Fig. 5A,B). In the rare cases in which axons cross onto the aggrecan, they grow markedly more slowly than on the favorable substrate and often stop growing altogether^11^. Here, neurons were treated with control or fidgetin siRNA nanoparticles at the time of plating, and then assessed for crossing 2 days later. Double-labeling IF for neuron-specific β-III-tubulin and fidgetin shows an increase in microtubule invasion into the growth cone, concomitant with the expected decrease in fidgetin (Fig. 5C,D). Seventy-seven axons were identified approaching the gradient and documented as either crossing or not crossing the aggrecan stripe; 35 were control siRNA treated cultures and 42 were fidgetin siRNA treated cultures. Of the control siRNA treated axons assessed, 29 (82.9%) did not cross and 6 (17.1%) crossed. Of the fidgetin siRNA treated axons assessed, 27 (64.3%) did not cross and 15 (35.7%) crossed. There was no statistically significant association between nanoparticle treatment and axons crossing. Knockdown of fidgetin exhibits a trend toward increasing the frequency of axonal crossing (Fig. 5E).

Data for axonal length of crossing axons were not normally distributed for fidgetin siRNA treated axons, as assessed by Shapiro-Wilk Test (p = 0.025), whereas control siRNA treated axons were normally distributed (p = 0.584). Control siRNA treated axonal length (n = 6) had a skewness of 2.298 (standard error = 0.845) and kurtosis of 5.388 (standard error = 1.741). Fidgetin siRNA treated axonal length (15) had a skewness of 0.482 (standard error = 0.580) and kurtosis of −1.542 (standard error = 1.121). The data do not appear normally distributed for either group, and therefore a non-parametric comparison of medians was performed. A Mann-Whitney U test was run to determine the difference between the crossing-axonal length of control and fidgetin siRNA treated axons. Axonal length for fidgetin siRNA treated crossing axons were statistically significantly longer (mean rank = 12.40) than control siRNA axons (mean rank = 4.80), U = 9, z = −2.488, p = 0.013. Therefore, when the axons from knockdown neurons did cross, their growth rate was over three times greater than control axons that crossed into aggrecan, and this effect was statistically significant (Fig. 5F).

## Discussion

**Figure 6:**
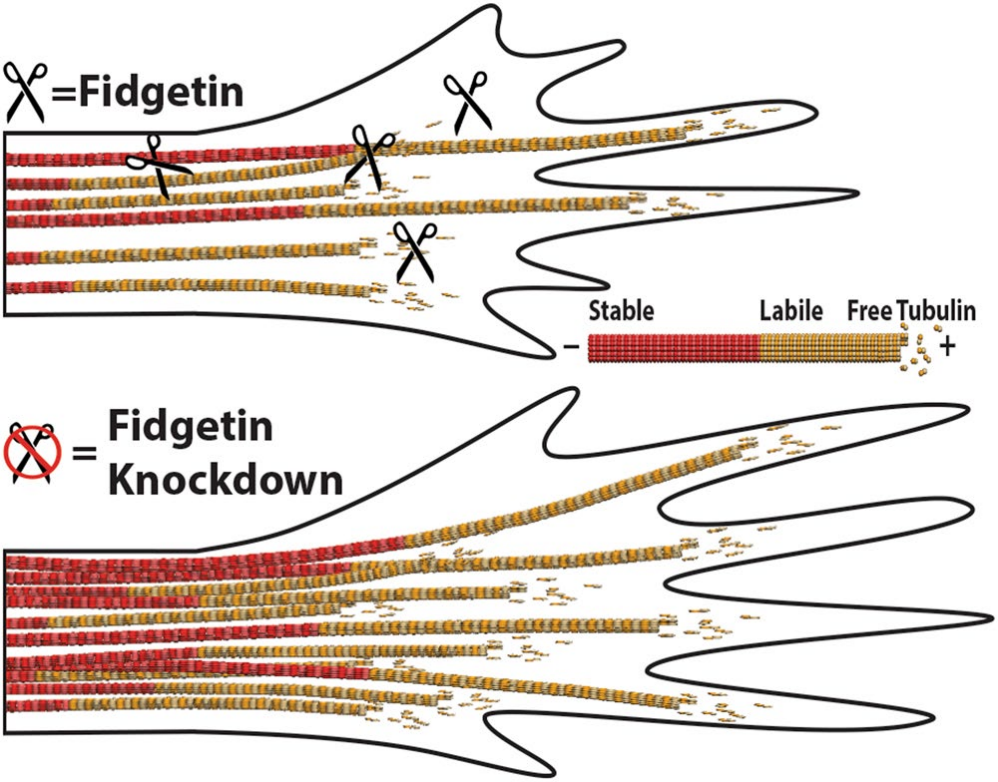
Schematic illustration of how fidgetin knockdown boosts axonal regeneration. Fidgetin severs axonal microtubules in the labile domain of the microtubule, and thus has the function of paring back the labile domains, tamping back their length. Experimental partial depletion of fidgetin enables elongation of the labile domains. Labile domains are especially enriched in the growth cone, and hence their elongation promotes invasion of microtubules into filopodia, which in turn promotes greater axonal growth, even through inhibitory molecules associated with nerve damage. Theoretically, axons with greater invasion of labile microtubule domains into their growth cones should be better equipped to navigate to their targets, both relative to injured axons and injured axons treated with microtubule-stabilizing drugs.

Using adult primary DRG cultures, a broadly accepted *in vitro* model for evaluating cell biological hypotheses relevant to nerve regeneration, we set out to test whether partial knockdown of fidgetin has potential for providing therapeutic benefit. We took the nanoparticle approach both out of necessity (because adult DRG cultures do not transfect well by conventional methods) and because the approach can be translated to future *in vivo* and clinical work. The results on the adult DRG neurons were consistent with those on the fetal cortical neurons transfected by nucleofection^9^, but the level of knockdown and the phenotype were somewhat more modest, with 19% increase in microtubule mass per unit length of axon, rather than 62%. Growth rates of axons were increased accordingly, and this was the case whether the axon grew on a favorable substrate or an unfavorable one composed of a growth-inhibitory protein of the glial scar tissue associated with spinal cord injury. Drug studies and tubulin acetylation studies confirmed that, like our earlier work on fetal cortical neurons, the increase in microtubule mass was due specifically to an increase in the labile microtubule fraction. This is consistent with fidgetin normally paring back the labile fraction by targeting regions of microtubules that are rich in tubulin that has not been post-translationally acetylated (Fig. 6). A greater level of knockdown theoretically could be achieved by manipulating the composition of the nanoparticles, but in fact, the best therapeutic is probably one that lowers the relevant protein modestly so as not to completely impede the normal work of that protein.

We posit that modest fidgetin knockdown offers a potentially superior microtubule-based approach for augmenting nerve regeneration compared to other microtubule-based approaches that have recently been tried. One of the challenges for the regenerating axon is navigation to its appropriate target tissue. Most treatments that enable axons to overcome inhibitory factors would also be detrimental to appropriate axonal navigation. This includes microtubule-stabilizing drugs as well as inhibitors of kinesin-5, a molecular motor that imposes growth-regulatory forces on the axon^11^. Axons navigate the best when they are richly endowed with labile microtubules extending into their distal regions^12, 13^, and thus fidgetin inhibition may provide an advantage over these other approaches in terms of enabling the regenerating axon to grow rapidly through inhibitory environments while being primed to navigate to its appropriate target.

At present, the most common experimental method for pursuing knockdown work in an animal model is to introduce RNA interference via a plasmid driven by a viral transduction system. Our nanoparticle approach may be a superior option; it is non-toxic, effective on cells, tissues and organs, and is controllable. The composition of the particles can be varied so that they release their load at different rates, so that treatment regimens can be refined accordingly. For example, it may be desirable to knock down a microtubule-related protein for a window of time that would enable a regenerating axon to grow through the glial scar, but only during the time required for that to happen. Such an approach may provide new hope for kinesin-5 as a target, if it could be knocked down only during a brief window of time so that its knockdown would not impede axonal navigation. Fidgetin, on the other hand, could be knocked down for longer periods of time through slower time-release of the siRNA from the nanoparticles because its knockdown would presumably assist in axonal navigation. Also, unlike the viral approach, which continues to express indefinitely, the nanoparticles would mitigate adverse off-target effects because such effects would be reversible after the siRNA load has been fully released and degraded.

In conclusion, we present fidgetin as a promising therapeutic target for nerve injury (Fig. 6), and we present a flexible nanoparticle-based platform as a promising mode of delivery for the siRNA (Fig. 1A).

## Methods

### RNA interference

A pool of siRNA consisting of four independent non-overlapping sequences for rat fidgetin was used in our previous study on rat fetal cortical neurons^9^. In that study, because of the lack of effective fidgetin antibodies, efficiency of knockdown was assessed by Western blot analysis of levels of ectopically expressed GFP-fidgetin, using a GFP antibody. Appropriate control experiments were conducted on the specificity of the siRNA pool, including confirmation that the identical phenotype was obtained when the four siRNA sequences were used individually. Here we used the same siRNA pool as in the previous work, in order to capitalize on the extensive controls done in that study. For preparation of the fidgetin siRNA-fused nanoparticles (or control non-specific siRNA-fused nanoparticles), a volume of five hundred microliters of Tetramethyl orthosilicate (TMOS) was hydrolyzed in the presence of 100 μl of 1 mM HCl by sonication on ice for 15 minutes, until a single phase formed^10^. The hydrolyzed TMOS (100 μl) was added to 900 μl of 20 μM of siRNA solution (either control or fidgetin pool) containing 10 mM phosphate, pH 7.4. The resulting gel, which formed within 10 minutes, was frozen at −80 °C for 15 minutes and lyophilized. siRNA-fused nanoparticles that had been re-suspended in water and sonicated were added to the culture medium at 2 μl/ml. Efficacy of fidgetin knockdown was ascertained by quantitative PCR and also qualitatively by IF-staining with a fidgetin antibody that we have found to be effective for IF (see below). Optical sections of identical thickness were obtained with a confocal microscope to compare fidgetin IF-staining levels in neuronal cell bodies. Some batches of siRNA-encapsulated nanoparticles proved ineffective at knockdown, and such batches were discarded.

### Cell culture

Primary cultures of DRGs were prepared by a modification of our previously published method^11^. DRGs were dissected from spinal cords of adult female rats (using protocols approved by Drexel University’s IACUC, and consistent with NIH regulations) and then exposed to 0.25% collagenase for 1 hour, followed by 0.25% trypsin for 15 minutes. Ganglia were then rinsed of the enzymes using Neurobasal A with 1% fetal bovine serum (FBS). Cells were suspended in culture medium containing Neurobasal A, B27, NGF, Glutamax, and Pen/Strep, as previously described^11^, and plated onto glass-bottomed 35 mm dishes in culture medium. The cell suspension was poured over a microsieve to remove debris. Prior to plating the cells, the glass-bottomed wells of culture dishes in which a 1 cm hole was covered with a glass coverslip were treated with poly-D-lysine, as previously described^9, 11^. DRG cultures were plated denser (10,000 cells/well) for microtubule analyses and sparser (2500 cells/well) for morphological analyses.

### Sample preparation

On the third day of culture, cultures were prepared for IF or qPCR. For IF on fidgetin, cultures were not pre-extracted but rather directly fixed with paraformaldehyde, then extracted in buffer containing TrixonX-100, and IF-stained with a commercially-available antibody (termed SC68343, obtained from Santa Cruz). For IF on microtubules, cultures were pre-extracted for 4 minutes in a microtubule-stabilizing buffer containing TritonX-100 to release free tubulin, and then fixed in a solution containing both paraformaldehyde and glutaraldehyde as previously described^14^. For qPCR, cultures were not pre-extracted and RNA was isolated using the RNAqueous-Micro Kit (AM1931) from Ambion^9, 11, 15^. qRT-PCR was performed as previously described^16^. Quantification of microtubule levels was conducted for IF using an antibody to β-III-tubulin (termed MMS-435P, obtained from BioLegend), which is neuron-specific. In some experiments, cultures were double-labeled for acetylated tubulin (with an antibody termed 6–11b-1, obtained from Sigma) as well as β-III-tubulin. β-III-tubulin IF-staining was used for both microtubule quantification experiments as well as for morphological analyses on axonal length. This was especially helpful for the DRG cultures, which are dominated by non-neuronal cells that can make distinguishing axons otherwise problematic.

### Microtubule quantification, stability and acetylation analyses

For quantification of microtubule levels in neurons, cultures were exposed for 0, 0.5, or 2 hours to nocodazole (2 mg/ml) or DMSO (vehicle control). For studies on the functional relevance of microtubule acetylation, some cultures were treated with tubacin (10 μM) or DMSO (vehicle control) during the second and third days of culture, and then subjected to morphological analyses. Most imaging was conducted using a Zeiss Observer microscope, 100X oil objective (for microtubule quantification) or 40x oil objective (for morphometry), Axiocam CCD, and Zen Blue software, except for confocal imaging, which was conducted with a Zeiss Pascal confocal microscope. For the microtubule quantification experiments, approximately 600–800 axons were imaged per dish (3 dishes per treatment condition). Using ImageJ, a region of interest (ROI) was traced around the axon and thresholding was performed to remove background signal and quantify the average mean gray value of fluorescence (0–255). Fluorescence intensity from the single-label β-III-tubulin IF-staining was calculated per unit length of axon. In other studies, for control and fidgetin siRNA nanoparticle treated cultures, the ratio of fluorescence intensity for acetylated to total tubulin was acquired for the axon by previously described methods^9^. Approximately 60 measurements were taken per dish, 3 dishes per treatment. In other experiments, the fluorescence intensity of acetylated tubulin was expressed as a ratio to total tubulin, as an independent indicator of microtubule stability.

### Morphological analyses

Morphological analyses were conducted on cultures treated with control siRNA nanoparticles or fidgetin siRNA nanoparticles, with or without tubacin. The average axonal length was quantified. In a separate set of experiments, the ability of axons to cross from a laminin substrate onto aggrecan was assessed, using a modified version of a previously reported assay^11, 17^. In brief, glass-bottomed dishes were coated with 0.1 mg/ml poly-D-lysine, rinsed thoroughly with water and allowed to air-dry. Strips (1 mm × 1 cm) of filter paper that had been soaked with 3 μl of aggrecan solution (prepared 75 μg/ml in water) were placed in the dried wells, and each strip was then allowed to dry, leaving stripes of aggrecan attached to the substrate upon the strip’s removal. Dishes were then coated with 10 μg/mL laminin. Adult DRG neurons were plated in thin lines of 250 cells on either side of the aggrecan stripes. Immediately after settling, cells were treated with medium containing either control or fidgetin siRNA nanoparticles. Cells were fixed after 48 hours, IF-stained for CS-56 (antibody to aggrecan, Sigma) and β-III-tubulin, and analyzed for axons that had approached within 10 μm of the stripes^11^. Axons that had turned so that a line drawn from the tip of the axon would point away from the stripe were defined as “not crossed,” whereas axons that had grown over the aggrecan border were counted as “crossed.” Axons grown from cell bodies that had landed within 20 μm of an aggrecan stripe or on the stripe itself were excluded.

### Statistics

Statistical analyses were conducted using IBM SPSS 24 and detailed with the corresponding data in the results section. Data are mean ± standard deviation, unless stated otherwise. All data were checked for normality using the Shapiro-Wilk test. Parametric data were assessed using the t-test to compare means and non-parametric data utilized the Mann-Whitney U test to compare medians. Two tailed tests were performed and all dependent values were continuous with dichotomous independent variables. A Chi-square test was performed to test for an association between categorical data.

### Data Availability

The datasets generated during and/or analyzed for the current study are available from the corresponding author on reasonable request.

## References

[1] Liu K, Tedeschi A, Park KK, He Z (2011). Neuronal intrinsic mechanisms of axon regeneration. Annu Rev Neurosci.

[2] Mar FM, Bonni A, Sousa MM (2014). Cell intrinsic control of axon regeneration. EMBO Rep.

[3] Hellal F (2011). Microtubule stabilization reduces scarring and causes axon regeneration after spinal cord injury. Science.

[4] Ruschel J (2015). Axonal regeneration. Systemic administration of epothilone B promotes axon regeneration after spinal cord injury. Science.

[5] Popovich PG, Tovar CA, Lemeshow S, Yin Q, Jakeman LB (2014). Independent evaluation of the anatomical and behavioral effects of Taxol in rat models of spinal cord injury. Exp Neurol.

[6] Baas PW (2014). Beyond taxol: microtubule-based strategies for promoting nerve regeneration after injury. Neural Regen Res.

[7] Baas PW, Ahmad FJ (2013). Beyond taxol: microtubule-based treatment of disease and injury of the nervous system. Brain.

[8] Matamoros AJ, Baas PW (2016). Microtubules in health and degenerative disease of the nervous system. Brain Res Bull.

[9] Leo L (2015). Vertebrate Fidgetin Restrains Axonal Growth by Severing Labile Domains of Microtubules. Cell Rep.

[10] Charafeddine RA (2015). Fidgetin-Like 2: A Microtubule-Based Regulator of Wound Healing. J Invest Dermatol.

[11] Lin S (2011). Inhibition of Kinesin-5, a microtubule-based motor protein, as a strategy for enhancing regeneration of adult axons. Traffic.

[12] Tanaka E, Ho T, Kirschner MW (1995). The role of microtubule dynamics in growth cone motility and axonal growth. J Cell Biol.

[13] Challacombe JF, Snow DM, Letourneau PC (1997). Dynamic microtubule ends are required for growth cone turning to avoid an inhibitory guidance cue. J Neurosci.

[14] Baas PW, Black MM (1990). Individual microtubules in the axon consist of domains that differ in both composition and stability. J Cell Biol.

[15] Liu K (2010). PTEN deletion enhances the regenerative ability of adult corticospinal neurons. Nat Neurosci.

[16] Barson JR (2017). Substance P in the anterior thalamic paraventricular nucleus: promotion of ethanol drinking in response to orexin from the hypothalamus. Addict Biol.

[17] Beller JA (2013). Comparison of sensory neuron growth cone and filopodial responses to structurally diverse aggrecan variants, *in vitro*. Exp Neurol.

